# Construction of a laparoscopic appendectomy model

**DOI:** 10.1590/0100-6991e-20243770-en

**Published:** 2024-07-26

**Authors:** JOSÉ NILO DE-LIMA, ARTHUR MENEZES DA SILVA, DAVI CASTRO FREIRE, ELLEN DAYANE DANTAS RODRIGUES, ANNYA MACEDO GOES, ADRIELY OLIVEIRA QUINTELA, FRANCISCO DUQUE DE PAIVA GIUDICE, JOSÉ GONZAGA DA-SILVA

**Affiliations:** 1 - Universidade Federal do Ceará, Faculdade de Medicina - Fortaleza - CE - Brasil; 2 - Universidade Federal do Ceará, Departamento de Cirurgia - Fortaleza - CE - Brasil; 3 - Coordenadora da Residência Médica do Hospital Universitário Walter Cantídio (HUWC). - Fortaleza - Ceará - Brasil; 4 - Universidade Federal do Ceará, Laboratório de Simulação Avançada - Fortaleza - CE - Brasil

**Keywords:** Education, Medical, Simulation Training, General Surgery, Educação Médica Continuada, Capacitação Profissional, Procedimentos Cirúrgicos Minimamente Invasivos, Exercício de Simulação

## Abstract

**Introduction:**

Appendectomy is the standard treatment for appendicitis, with the laparoscopic technique offering benefits like lower infection rates and quicker recovery. However, residents often have their first practical experience with the procedure on real patients, increasing surgical risks. In this context, medical simulation emerges as a crucial methodology, allowing professionals to experience a variety of scenarios while preventing harm to patients. The objective of this study is to describe the production of an “ex-vivo” simulation model for laparoscopic appendectomy.

**Methodology:**

Cold ceramic structures were used to manually shape the anatomical model of the appendix, ensuring its rigidity. On this model, we poured materials to create a flexible mold using acetic silicone. Once the mold was made, we filled it with thermo-moldable styrene polymer rubber, along with dye, and fused it at a specific temperature.

**Results:**

This process resulted in the manufacture of a piece that simulates the appendix, being tear-resistant and suturable, faithfully replicating the structure and characteristics of a human organ. The low weight of the materials facilitates transport, allowing them to be reproduced and used in various situations, from training in hospital settings to universities. The model is applicable in didactic simulations with medical students, residents, and surgeons. Its ease of production and low cost contribute to the practices being repeatable, ensuring a better development of surgical skills.

**Conclusion:**

This work not only contributes to the advancement of medical simulation but also highlights the importance of innovative and collaborative solutions in improving medical education and promoting patient safety.

## RESUMO

**Introdução:**

A simulação médica emerge como uma metodologia crucial, permitindo que profissionais experimentem uma variedade de cenários, tanto de êxito quanto de falha, identificando fragilidades e condições inseguras, com o intuito de evitar danos aos pacientes. Assim, o objetivo desse estudo é descrever a produção de um modelo de simulação em apendicectomia videolaparoscópica “ex-vivo”.

**Metodologia:**

Foram utilizadas estruturas de cerâmica fria para moldar manualmente o modelo anatômico do apêndice, garantindo sua rigidez. Neste modelo, utilizou-se materiais para criar um molde flexível usando silicone acético. Feito o molde, o preencheu-se com borracha de polímero de estireno termomoldável, junto com corante, e fundindo em uma temperatura específica.

**Resultados:**

Esse processo resultou na confecção de uma peça que simula o apêndice, sendo resistente ao rasgo e suturável, replicando fielmente a estrutura e características de um órgão humano. O baixo peso dos materiais facilita o transporte, permitindo que sejam reproduzidos e utilizados em diversas situações, desde treinamentos em ambientes hospitalares até universidades. O modelo é aplicável em simulações didáticas com estudantes de medicina, residentes e cirurgiões. Sua facilidade de produção e baixo custo contribuem para que as práticas sejam repetíveis, garantindo um melhor desenvolvimento das habilidades cirúrgicas.

**Conclusão:**

O trabalho contribui para o avanço da simulação médica e destaca a importância de soluções inovadoras e colaborativas na melhoria da educação médica e na promoção da segurança do paciente.

## INTRODUCTION

For more than a century, appendectomy has been the standard treatment for appendicitis worldwide. The approach by laparoscopy, introduced by Semm in 1983, almost a century after McBurney’s open surgery in 1891, has grown exponentially^1^. Currently, it is the preferred method due to its benefits, such as less infection, less pain, shorter length of hospital stay, and faster return to activities^1^. The high costs associated with laparoscopic appendectomy training models available on the market pose a significant challenge for many hospitals, especially those with limited financial resources. As a result, residents are often compelled to acquire their initial skills directly from real patients, which not only increases risks during surgery but also compromises patient safety^2^. 

In this context, simulation emerges as a crucial methodology, allowing residents to experiment with a variety of scenarios, both success and failure, identifying weaknesses and unsafe conditions, to avoid harm to patients. High-risk and high-responsibility professions have adopted realistic simulation as a training tool, aiming to prevent serious incidents through timely corrective practice. 

The concept that simulation is restricted to technological and robotic environments, which are inaccessible to most training grounds, is being revised. In its place, the manufacture of handmade, low-cost models and simulators is emerging, which allow training in specific procedures, including simulations of individual or team scenarios, aimed at developing non-technical skills such as communication, decision-making, leadership, teamwork, and feedback. The incorporation of real patients in the educational process, in addition to posing ethical considerations, can potentially compromise the learning process due to the embarrassment or discomfort experienced by the resident, as well as the complexity of the situations presented, which can be difficult to understand and manage due to the lack of previous experience. On the other hand, the use of simulated models plays a key role in training residents, enabling them to interact with real patients more effectively. This is achieved through the opportunity to repeat procedures, accompanied by feedback to identify and review errors and successes^3^. 

In this sense, this study describes the production of an ex vivo simulation model in laparoscopic appendectomy, with the objective of encouraging the replication of this prototype in hospital units that teach this technique in medical residency. It is expected that the use of this model will improve technical performance in the operating room compared with traditional in vivo surgical training^4^.

## METHODS

### Assembling the simulator

We used cold ceramic structures (commercially known as Biscuit) to manually model the anatomical replica of the appendix, ensuring its rigidity. On top of this model, we created a flexible acetic silicone mold. Once the mold was made, it was filled with thermo-moldable rubber of Styrene Polymer and dye, melting it later. The Styrene Polymer is inserted into the flexible mold until it is completely cooled, solidifying and becoming an appendix model ready for use in surgical practices, as in the figure below. Table 1 brings average values of the products used.

**Table 1:** Average values of the materials used.

Material	Value (average)*
Biscuit 500g	R$ 20.00
Acetic Silicone 256g (tube)	R$ 26.00
Styrene Polymer 800g	R$ 82.00

**Figure 1:**
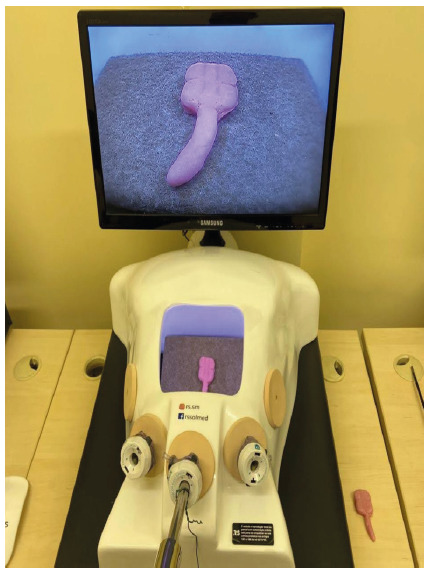
Videolaparoscopy simulator (RS Medical Solutions) with an appendix model inserted and supported by spongy material.

**Figure 2:**
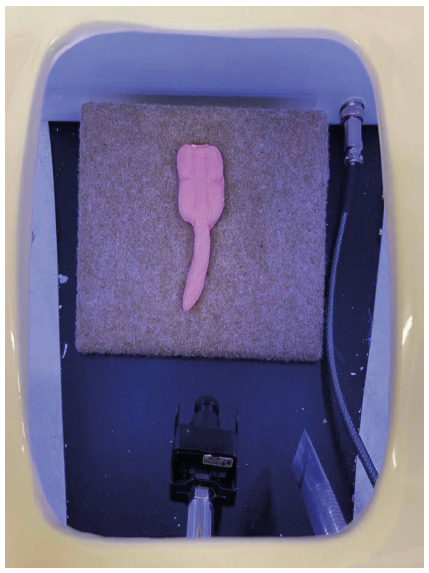
Simulator model of the appendix supported by a spongy support.

The laparoscopic model was made using the mannequin and materials from RS Soluções Médicas to simulate the laparoscopic system with an LCD screen (Figures 1 and 2).

## RESULTS

This process resulted in the manufacture of a model that simulates the appendix, resistant to tearing and suturable, faithfully replicating the structure and characteristics of a human organ.

The materials used are totally synthetic, so they have no risk of infection, and their low weight makes it easy to transport, allowing them to be reproduced and used in various situations, from training in hospital spaces to universities. The appendectomy model can be applied for practical teaching with undergraduate students, aspiring surgical physicians, residents, and surgeons, and is indicated for use in didactic simulations, supervised by experienced advisors. 

During simulation, it is possible to evaluate basic techniques, highlighting the management of surgical instruments, such as correct handling of grips, notion of depth in a virtual space, up to the making of a surgical knot in an endoscopic model.

The support used is structured with the mold of a human abdomen, which ensures strategic learning regarding the laparoscopic technique in the abdominal region, since the anatomical landmarks for the insertion of the devices are previously delimited. We highlight the role of the support in simulating the external anatomical model and in promoting support for the instruments used endoscopically, allowing the introduction to the functioning of minimally invasive surgery, its instruments, and its anatomical landmarks.

## DISCUSSION

Appendectomy is a surgical procedure widely performed on an emergency basis, especially in pediatric, adolescent, and young adult populations. Currently, the laparoscopic approach is preferred due to its minimally invasive nature, associated with a lower incidence of complications, reduced recovery time and hospital stay, as well as a lower rate of surgical wound infection and reduced postoperative pain^5^.

The use of simulators aims to reproduce practical situations found in daily practice, with the main purpose of training professionals in various areas of knowledge, preparing them to face critical situations and to acquire the necessary technical skills. This teaching method, widely adopted in Surgery residency programs, aims to train residents before performing procedures on real patients^6^. Learning through simulators is divided into three stages: the first encompasses the rapid acquisition of manual skills, followed by the consolidation of learning in the second phase, and finally the retention of the skills acquired in the third phase. Repeated practice results in a continuous improvement in the learning curve until a point of stabilization is reached^7^.

Laparoscopic appendectomy simulation is justified as part of training in non-clinical settings, minimizing the trauma and risks of complications associated with inexperience with the procedure. In addition, the ability to perform laparoscopic surgeries is increasingly in demand and is considered a fundamental competence for surgeons^8^. 

A study conducted in Scotland, which included an economical and realistic model of appendectomy, revealed that a simulation with 49 surgeons, both novice and experienced, obtained evidence of validity, since it contributed to the evaluation of the transferability of skills from the simulator to the clinical environment, while the existence of repetition and individualized feedback during teaching helps in learning^9^.

Therefore, the construction of more simulated models, especially aimed at training physicians beginning in laparoscopic surgery, is essential to promote a more efficient learning curve, reduce potential risks for patients involved in training procedures, and facilitate access to innovative teaching methodologies for surgeons in training. 

It is also important to consider that each appendix model can only be used once. In this sense, the amount needed for its practical application depends on the number of simulations to be performed. The number of lines and silicone models must be thought out to supply a sufficient amount according to the expected repetitions.

The model does not have the purpose of anatomical or pathological reproduction of the gastrointestinal tract, with the limitation of not being able to reproduce pathological findings related to the inflammatory process, anatomical variations, and risks associated with perforation of the large intestine or peripheral organs. However, these limitations are partially overcome by examining the dexterity of handling the synthetic organ, without moving it beyond a previously delimited space, and by observing the efficacy of the technique of performing the surgical knot.

## CONCLUSIONS

This work not only contributes to the advancement of medical simulation but also highlights the importance of innovative and collaborative solutions in improving medical education and promoting patient safety. By providing a prototype for the ongoing development of surgical skills and clinical practices, this simulation model has the potential to transform the way learning surgeons are trained and, in turn, improve clinical outcomes for patients around the world. The replication and implementation of this model can bring significant benefits to the teaching and practice of laparoscopic appendectomy, as well as serve as a basis for future research and development in the field of medical simulation.
